# A Smartphone Application for Personal Assessments of Body Composition and Phenotyping

**DOI:** 10.3390/s16122163

**Published:** 2016-12-17

**Authors:** Gian Luca Farina, Fabrizio Spataro, Antonino De Lorenzo, Henry Lukaski

**Affiliations:** 1 Section of Clinical Nutrition and Nutrigenomic, Department of Biomedicine and Prevention, University of Rome “Tor Vergata”, Rome 00173, Italy; gianluca.farina@students.uniroma2.eu (G.L.F.); fabrizio.spataro@students.uniroma2.eu (F.S.); 2 Department of Kinesiology and Public Health Education, University of North Dakota, Grand Forks, ND 58202-7166, USA; henry.lukaski@email.und.edu

**Keywords:** body composition assessment, mobile health, weight management

## Abstract

Personal assessments of body phenotype can enhance success in weight management but are limited by the lack of availability of practical methods. We describe a novel smart phone application of digital photography (DP) and determine its validity to estimate fat mass (FM). This approach utilizes the percent (%) occupancy of an individual lateral whole-body digital image and regions indicative of adipose accumulation associated with increased risk of cardio-metabolic disease. We measured 117 healthy adults (63 females and 54 males aged 19 to 65 years) with DP and dual X-ray absorptiometry (DXA) and report here the development and validation of this application. Inter-observer variability of the determination of % occupancy was 0.02%. Predicted and reference FM values were significantly related in females (R^2^ = 0.949, SEE = 2.83) and males (R^2^ = 0.907, SEE = 2.71). Differences between predicted and measured FM values were small (0.02 kg, *p* = 0.96 and 0.07 kg, *p* = 0.96) for females and males, respectively. No significant bias was found; limits of agreement ranged from 5.6 to −5.4 kg for females and from 5.6 to −5.7 kg for males. These promising results indicate that DP is a practical and valid method for personal body composition assessments.

## 1. Introduction

Obesity is a major public health problem in the US and worldwide [1,2]. Despite intensive and multi-focal efforts to attenuate the rate of increase of obesity, this problem has persisted. Personal health assessments, a form of self-monitoring [3], empower individuals to engage in behaviors and lifestyles that can mitigate risk of development of chronic diseases and improve quality of life [4,5]. The use of personal health assessments is a burgeoning approach in the management of overweight and obesity [6,7]. A practical attraction of personal health assessment is the privacy and convenience of performing these activities at home.

Consumer devices enable an individual to participate in personalized health assessments for weight management. A growing area of interest is the determination and monitoring of personal biometrics and body phenotype including body composition and size. Simple measures such as body weight, height, waist, and hip circumferences are desirable because of their associations with risk factors for metabolic and chronic diseases [5]. These measurements, however, can be time-consuming and require training and proficiency to provide reliable and useful information for personal and clinical use. Self-monitoring of body weight and various biometrics are effective in the promotion of successful weight loss, weight maintenance, and weight regain prevention [8,9,10]. Assessments of body fat during weight loss intervention are important to enable the preservation of lean body mass [11].

Personal body composition assessments can be completed at home with bioelectrical impedance-based assessments (BIA), such as foot-to-foot and hand-to-hand impedance scales or even smaller finger-to-finger devices [12,13]. Although widely publicized and used as fat analyzers, BIA devices are limited in accuracy in terms of assessing body fat largely due to several faulty assumptions such as a steady hydration of the lean tissues [13,14,15]. Alternatively, whole-body optical scanning devices to estimate body volume, size, and regional body circumferences are emerging [16,17,18,19]. They yield reasonably accurate measurements but require relatively costly and cumbersome equipment, large space requirements and lighting that makes them impractical for routine personal use [20]. 

We evaluate a novel application for smart phones that provides a simple and cost-effective method of determining body fat for an individual. It utilizes a smartphone built-in camera to obtain digital whole-body images to estimate human body composition. The objectives of this research are to develop and validate a digital image photography model to assess human body composition. We tested the hypothesis that digital photography with a smart phone is a valid method to estimate the body fat of adults.

## 2. Methods and Materials

Caucasian women and men, varying widely in age and body mass index, volunteered to participate in this study, which was conducted at the Department of Physiology, University of Tor Vergata in Rome, Italy. Each prospective participant underwent a clinical examination and completed a health questionnaire to establish the absence of an unhealthy condition prior to participation. This study was approved by the Institutional Review Board of the University of Tor Vergata. Each participant provided written informed consent prior to participation in any testing.

### 2.1. Digital Photography

The operational principle of digital photography (DP) is that the surface of any digitally acquired image encased within a background can be computed by its occupation ratio within a digitally constructed virtual frame. Quantification of the number of pixels within the image of a body is expressed as a percentage of the total number of pixels in the framed background. It requires a clear and reliable discrimination of the pixels constituting the image of the body from the pixels determining the background. The ratio of the image pixels is expressed as a percentage of the total background pixels and termed the percent occupation by the image. The surface of an object or any of its components is computed from its frame percent occupation. Large and small occupation percentages are calculated into individual sagittal section surface areas that relate to volumes and can be transformed into body composition variables. 

### 2.2. Procedure

Each volunteer wore light, non-compressing underwear with or without light footwear and stood showing either lateral side in front of a homogeneous white background to obtain a significant contrast. With the head in a horizontal plane, the individual stood upright and fully extended the arms down alongside the body with feet and legs touching and aligned sagittal to the camera to provide a lateral profile of the body. A second individual directed the hand-held smart phone camera with the lens at the middle of the standing height of each subject. The distance from the camera to the subject was variable because of inter-individual differences in standing height and the requirement to surround the whole-body image within a minimal area of the background. Normal illumination in the room was adequate to allow for the capture of a clear and focused picture without flash.

**Figure 1:**
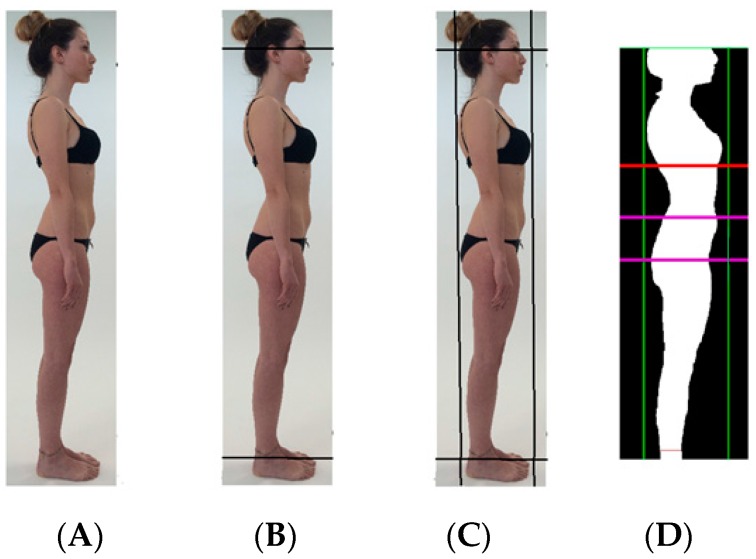
Images showing the sequence of operator-positioned and software-determined anatomical landmarks used to condition a digital photography lateral image. (**A**) Uploaded digital image of lateral surface of an individual; (**B**) operator-positioned horizontal lines at the level of the eyes and ankles; (**C**) operator-positioned vertical lines at widest protuberance of breast and hip; (**D**) software-determined horizontal lines at thorax, belly, and hips.

An operator uploaded the image (Figure 1A) and then framed the image by using software that provides an outline of the body to be conditioned with color to contrast the subject image from the background of a different color. The operator first positioned horizontal lines through the eyes and malleoli of the ankles (Figure 1B), and next inserted vertical lines along the widest anterior and posterior of the image (Figure 1C). An algorithm utilized a complex evaluation of the background pixel digits and allocated to that homogeneous pixel a value equivalent to black; the algorithm next allocated to the subject body image (e.g., non-background) pixels a value equivalent to white. This process represents the conditioning of an individual digital photograph with additional horizontal lines drawn by the proprietary software at the thorax, belly, and hips (Figure 1D). The determination of the populations of black and white pixels enables computation of the respective occupation percentages, specifically the individual image surface area.

### 2.3. Development of Prediction Model for Body Fat

One hundred seventeen healthy adults had lateral whole-body digital images with either Android version 4.2.2 on a Huawei G730 smart phone (resolution 540 × 960 pixels or 51.8 megapixels) or iOS 9.2 on an iPhone 5s (resolution 1136 × 640 pixels or 72.7 megapixels). The phones were randomly utilized to obtain digital images that were scaled by the software to a standard resolution of 5 megapixels. One operator performed all of the digital photographic images and stored all digital images to the memory of the smart phone. Shape recognition and image conditioning after the software-driven pixel count was performed on all digital images uploaded from the memory of the smart phone. Reference body composition was determined with dual X-ray absorptiometry (DXA; GE model LUNAR iDXAnCore s/n 200278; Rome, Italy) using software version General Electric 14.10.022.

Gender-specific prediction models for body fat were developed using multiple regression analyses. Independent variables included body weight, height, and lateral surface image occupancy from each conditioned image.

### 2.4. Statistical Methods

Descriptive data are expressed as mean ± SD. Estimates of inter-operator precision or reproducibility of image conditioning are reported as average values (±SD) and mean differences.

We used FM values determined from individual DXA scans as the dependent variable to develop prediction equations using forward step-wise regression with height, weight, image occupancy, and linear measurements obtained from digital images as independent variables. Gender-specific prediction equations were developed to account for known differences in FM and adipose tissue distribution between adult males and females [21,22]. For comparison of the prediction models, criteria included the highest adjusted R^2^ value and the lowest root mean square as a measure of precision [23]. The precision and robustness of the prediction equations were also assessed by calculating the PRESS statistic, which is the predicted residual sum of squares that can be used as a measure of predictive power when one sample is used to develop and validate a regression equation; it is an indicator of internal cross-validation strategy [24].

Measured and predicted FM values were compared with a paired *t*-test. A Bland–Altman plot was used to determine bias and limits of agreement for the derived prediction model [25]. 

Statistical analyses were performed using SYSTAT version 10 (Systat Corporation; San Jose, CA, USA), although the PRESS residuals and statistics were obtained using the PROC REG procedure with PRESS Statistics in SAS version 9.2 (SAS Institute, Inc., Crary, NC, USA).

## 3. Results

### 3.1. Inter-Operator Variability in Digital Image Conditioning

Three novice operators individually performed one conditioning of 20 identical digital images (10 males and 10 females) to assess the range of precision of assessment of percent occupancy, and verify the limit of agreement. The operators determined similar (*p* = 0.968) areas of occupancy (30.65% ± 3.6%, 30.74% ± 3.7%, and 30.71% ± 3.7%) with average differences between operators ranging from 0.033% to 0.097% that were not different than 0 (*p* = 0.908). Coefficients of determination (R^2^) for comparisons among operators for individual estimates of occupancy ranged from 0.972 to 0.996 (*p* < 0.0001) with an average difference of 0.02% among operators (*p* = 0.86).

### 3.2. Development and Validation of Prediction Model for Fat Mass

Table 1 summarizes the characteristics of the participants. Table 2 describes the gender-specific regression models to predict FM from DP. The prediction equation for females includes the lateral surface areas including the lower abdomen and hips, whereas the prediction model for males includes the same area as well as the upper abdomen and entire lateral surface area.

**Figure 2:**
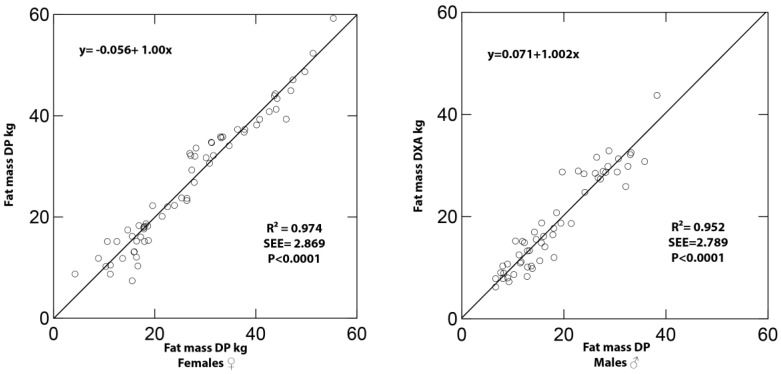
Plots of dual X-ray absorptiometry (DXA)-measured and digital image photography (DP)-predicted fat mass (FM) values of females (**left**) and males (**right**).

The DXA-determined and DP-predicted FM values were similar in the female (26.91 ± 12.74 and 26.97 ± 12.41 kg, respectively; *p* = 0.714) and male (18.79 ± 9.28 and 18.72 ± 8.83 kg, respectively; *p* = 0.838) groups. Within each gender group, measured and predicted FM values were significantly correlated (R^2^ = 0.991 and 0.982; *p* < 0.0001) with concordance correlation coefficients of 0.974 and 0.952 for males and females (*p* < 0.0001), respectively. The predicted and measured FM values were distributed linearly in the male and female samples with slopes similar to 1.0 and intercepts not different than 0 (Figure 2).

**Figure 3:**
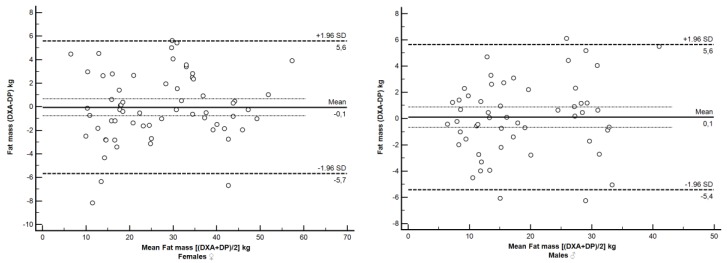
The Bland–Altman plots to illustrate the differences between individual dual X-ray absorptiometry (DXA)-measured and digital image photography (DP)-predicted fat mass (FM) as a function of the mean values of females (**left**) and males (**right**). Linear regression line describes the bias with 95% confidence intervals (1.96 SD) shown.

Figure 3 shows the distribution of the differences between measured and predicted FM values as a function of increasing FM. The differences between measured and predicted FM values were not different than 0 (−0.06 kg, *p* = 0.81; and 0.11 kg, *p* = 0.78) for females and males, respectively, with no proportional bias in the difference values as a function of the average FM values (r = 0.12, *p* = 0.86). The limits of agreement (mean ± 1.96 SD) ranged from 5.6 to −5.4 kg and from 5.6 to −5.7 kg for females and males, respectively.

## 4. Discussion

Behavioral approaches that utilize self-monitoring are a basic component of weight management programs with regular monitoring of body weight associated with successful weight loss and prevention of weight regain after weight loss [26,27]. Attributes of successful self-monitoring program include selection of measurements that are simple, are easy to perform, are not costly, use commonly available equipment, are performed accurately and privately, yield meaningful information for the individual, and may be shared with a health care provider. The present study met these criteria by using a camera of a common cellular phone to capture a digital image of the lateral surface of the body that was conditioned for analysis with proprietary software and yielded body surface estimates of regions epidemiologically associated with an increased risk of cardiometabolic disease that were used to estimate body fat in adults. It developed and validated a model in apparently healthy adults that accurately estimated FM, as compared to DXA FM values, in the range of 10–55 kg with no bias.

Traditional anthropometric measurements of body size and shape, including body mass index (BMI), various girths, body segmental circumferences, and mid-sagittal diameter are surrogates for adiposity and are associated with graded risks of cardio-metabolic disease [22,28]. Photonic imaging of the body with lasers, lights and cameras enables the generation of three-dimensional (3D) body outline images or shapes [29]. These methods eliminate the need for trained and certified anthropometrists but require costly and sophisticated data acquisition equipment, computerized algorithms to reconstruct body topography, and large space requirements [21,29]. Although originally developed for the clothing industry, national surveys used 3D imaging systems and observed differences in body phenotypes including differences in female and male shape patterns, surface area and volumes within the same BMI range [30], age-dependent effects on shape at a given BMI [30], and ethnic differences in shape [31]. Daniels et al. [32] reported that body segment volumes increased with increasing BMI and these BMI-related patterns of increase varied among different body segments. 

Emerging clinical applications include assessments of body phenotype and composition. Wells et al. [33] reported no difference in estimates of body volume of adults using 3D photonic imaging, whole-body air displacement plethysmography (ADP), and hydrodensitometry (HD) but wide LOA (>2 L) for optical imaging that equated to 20% variability in the estimation of body fat. Wang et al. [16] found that 3D imaging significantly overestimated (0.5 L) body volume compared to HD with no apparent differences in estimated body fat. Body fatness predicted with 3D imaging was significantly correlated with HD reference values with a wide distribution of the values (SEE = 7.95%). Garlie et al. [18] also reported no differences in body fat values among military personnel measured with 3D optical imaging, DXA, and military-specific anthropometric models. They noted, however, a higher concordance correlation coefficient for 3D imaging compared to anthropometry rather than to 3D imaging compared to DXA (0.96 vs. 0.74). Ng et al. [33] found significant correlations between 3D and reference measures of waist circumference, hip circumference, body surface area, and volume in adults. Predictions of body composition using 3D-determined circumferences were strongly related to DXA reference values of FM and fat-free mass with good precision (SEE = 2.5 and 2.2 kg, respectively).

Awareness of the adverse effects of increased centralized adipose tissue (AT) as a risk factor for cardio-metabolic disease prompted observational studies that utilized 3D imaging to assess regional AT. Lee et al. [34] reported that inclusion of the 3D-determined waist-to-hip ratio significantly improved multiple regression equations to predict MRI-estimated visceral AT but neither total abdominal AT nor subcutaneous AT. They also used 3D imaging to assess fat patterning of adults [35]. Compared to the usual demographic information (e.g., gender, age, and ethnicity) and standard anthropometric measurements (e.g., weight, height, and waist circumference), the inclusion of 3D body image data (e.g., regional volumes, circumferences and sagittal thicknesses) improved the precision, relative to DXA, to 2 kg and 0.2% for android and 3.2 kg and 0.4% for gynoid AT mass and % fat, respectively. 

An alternative to the costly 3D systems is two-dimensional (2D) imaging that uses frontal and/or lateral images obtained from a standard digital camera and software to characterize body shape. Validation of 2D hip, waist, neck, and trunk circumferences with standard anthropometric measures showed high correlations (R^2^ = 0.94 to 0.96) [36]. Stewart et al. [37] successfully used body shapes from 2D DP and 3D images in assessment of perception and dissatisfaction of the body images of individuals diagnosed with disorder eating behaviors. Xie et al. [19] generated active shape models (2D silhouettes) of children from modified whole-body DXA scans and compared the strength of the multiple regression equations that predicted percent body fat from standard demographic data and select 2D sites. For boys, the 2D model accounted for more variation in the prediction of body fat than the demography-based prediction model (R^2^ = 0.728, RMSE = 3.12% compared to R^2^ = 0.457, RMSE = 4.41%, respectively), whereas the 2D model accounted for similar variability in estimating body fat as the demography model for girls (R^2^ = 0.586, RMSE = 3.93% compared to R^2^ = 0.606, RMSE = 3.80%, respectively).

The present study provides the first validation of the 2D DP method to assess FM in adults with a wide range of body fat. Comparisons of DP-predicted and DXA-determined FM showed no significant differences between the methods with variability (SEE) of 2.83 and 2.71 kg for females and males, respectively, that is similar (2.4 kg) for a recent 3D imaging model [33].

These preliminary findings should be followed with future research to evaluate the present prediction model in adults with a wide range of BMI and different ethnic groups. Moreover, studies should ascertain the validity of this method during weight loss. Importantly, individuals who plan to utilize self-monitoring of body fat should consult their care provider for guidance.

Advancing technology provides a unique opportunity to enable healthy weight management. Smart phones offer a practical platform for personal health assessment and self-monitoring [38,39] because they can overcome some limitations of traditional weight loss and maintenance programs while reducing the cost and burden on patients and health care providers. Findings of the present study are promising for the use of a smart phone application to monitor body fat.

## Figures and Tables

**Table 1:** Physical characteristics of 117 study participants. Values are mean ± SD (range of values).

	Females	Males
n	63	54
Age, year	38.7 ± 13.8	32.5 ± 9.8
	(19 to 65)	(19 to 54)
Weight, kg	70.9 ± 15.6	82.0 ± 13.2
	(41.8 to 108.7)	(63.4 to 108.4)
Height, cm	162.7 ± 6.1	178.0 ± 7.7
	(152.0 to 174.9)	(163.0 to 194.5)
BMI ^a^, kg/m^2^	43.8 ± 12.6	62.8 ± 16.7
	(16.1 to 40.4)	(19.4 to 37.1)
Fat-free mass ^b^, kg	43.8 ± 12.6	62.8 ± 16.7
	(31.9 to 62.8)	(47.4 to 80.3)
Fat mass ^b^, kg	27.2 ± 12.7	19.2 ± 10.0
	(7.4 to 59.4)	(6.2 to 44.6)
Body fat, %	36.6 ± 10.8	22.5 ± 8.9
	(12.3 to 54.5)	(9.6 to 44.9)

^a^ Body mass index; ^b^ Dual X-ray absorptiometry.

**Table 2:** Multiple regression equations to predict body fat mass (FM) of 117 healthy adults.

Females: FM = 18.545 − 0.312 HT + 0.653 WT + 4.522 LOWERABD_HT
Males: FM = 56.602 + 0.799 PCTTOTAL − 0.063 SURFUP + 25.366 LOWERABD_HT

HT = height in cm; WT = weight in kg; LOWERABD = the surface (cm^2^) of the lateral surface between the lines drawn by the APP at the belly and the hip (Figure 1C); SURFUP = the surface (cm^2^) of the lateral section between the line at the belly drawn by the APP and the operator-drawn line at the eyes; PCTTOTAL = the percent of occupation of the entire lateral surface from ankle to eyes (Figure 1B).
