# Correction: Farina, G.L., et al. A Smartphone Application for Personal Assessments of Body Composition and Phenotyping. *Sensors* 2016, *16*, 2163

**DOI:** 10.3390/s17030434

**Published:** 2017-02-23

**Authors:** Gian Luca Farina, Fabrizio Spataro, Antonino De Lorenzo, Henry C. Lukaski

**Affiliations:** 1 Section of Clinical Nutrition and Nutrigenomic, Department of Biomedicine and Prevention, University of Rome “Tor Vergata”, Rome 00173, Italy; gianluca.farina@students.uniroma2.eu (G.F.); fabrizio.spataro@students.uniroma2.eu (F.S.); 2 Department of Kinesiology and Public Health Education, University of North Dakota, Grand Forks, ND 58202-7166, USA; henry.lukaski@email.und.edu

The authors wish to make the following corrections to Table 1 of their paper [1]:

As you may notice in the former Table 1, shown here, the mean values for BMI are 43.8 ± 12.6 for females and 62.7 ± 16.7 for males that are identical to the values for fat-free mass in the line below.

**Table 1:** Physical characteristics of 117 study participants. Values are mean ± SD (range of values).

	Females	Males
n	63	54
Age, year	38.7 ± 13.8	32.5 ± 9.8
	(19 to 65)	(19 to 54)
Weight, kg	70.9 ± 15.6	82.0 ± 13.2
	(41.8 to 108.7)	(63.4 to 108.4)
Height, cm	162.7 ± 6.1	178.0 ± 7.7
	(152.0 to 174.9)	(163.0 to 194.5)
BMI ^a^, kg/m^2^	43.8 ± 12.6	62.8 ± 16.7
	(16.1 to 40.4)	(19.4 to 37.1)
Fat-free mass ^b^, kg	43.8 ± 12.6	62.8 ± 16.7
	(31.9 to 62.8)	(47.4 to 80.3)
Fat mass ^b^, kg	27.2 ± 12.7	19.2 ± 10.0
	(7.4 to 59.4)	(6.2 to 44.6)
Body fat%	36.6 ± 10.8	22.5 ± 8.9
	(12.3 to 54.5)	(9.6 to 44.9)

^a^ Body mass index; ^b^ Dual X-ray absorptiometry.

The BMI values (mean ± SD) shown in Table 1 are erroneous since the range of values is a minimum of 16.1 to a maximum of 40.4 with a reported mean of 43.8 for females, and a minimum of 19.4 to a maximum of 37.1 with a mean of 62.8 for males. Therefore, the listed and incorrect mean values exceed the maximum values for females and males, respectively. The mean value for the BMI should be 26.8 ± 5.8 for females and 25.9 ± 4.2 for males. Therefore we want to replace the above table with this correct version shown below:

**Table 1:** Physical characteristics of 117 study participants. Values are mean ± SD (range of values).

	Females	Males
n	63	54
Age, year	38.7 ± 13.8	32.5 ± 9.8
	(19 to 65)	(19 to 54)
Weight, kg	70.9 ± 15.6	82.0 ± 13.2
	(41.8 to 108.7)	(63.4 to 108.4)
Height, cm	162.7 ± 6.1	178.0 ± 7.7
	(152.0 to 174.9)	(163.0 to 194.5)
BMI ^a^, kg/m^2^	26.8 ± 5.8	25.9 ± 4.2
	(16.1 to 40.4)	(19.4 to 37.1)
Fat-free mass ^b^, kg	43.8 ± 12.6	62.8 ± 16.7
	(31.9 to 62.8)	(47.4 to 80.3)
Fat mass ^b^, kg	27.2 ± 12.7	19.2 ± 10.0
	(7.4 to 59.4)	(6.2 to 44.6)
Body fat%	36.6 ± 10.8	22.5 ± 8.9
	(12.3 to 54.5)	(9.6 to 44.9)

^a^ Body mass index; ^b^ Dual X-ray absorptiometry.

We apologize for any inconvenience these changes have caused to readers. These changes do not affect the results of this research. The manuscript will be updated and the original will remain online on the article webpage. 
